# Role of transgenerational transmission of trauma in development of schizophrenia: A case report of a patient whose parents survived genocide in Srebrenica

**DOI:** 10.1192/j.eurpsy.2023.1316

**Published:** 2023-07-19

**Authors:** N. Zigic, E. Becirovic, M. Mirkovic-Hajdukov, N. Aljukic, H. Löffler-Stastka

**Affiliations:** 1 Department of Psychiatry, University Clinical Center Tuzla, Bosnia and Herzegovina, University Clinical Center Tuzla, Tuzla, Bosnia and Herzegovina; 2 Department of Psychoanalysis and Psychotherapy, Medical University Vienna, Vienna, Austria

## Abstract

**Introduction:**

Developmental predisposition to schizophrenia can be a consequence of early experienced traumas. Transgenerational trauma is process in which traumatic experience of one generation is passed on to the next generation.

**Objectives:**

To show connection between transgenerational transmission of trauma and development of schizophrenia.

**Methods:**

Psychiatric interview, psychological testing.

**Results:**

Patient G.E. age 29, admitted to Psychiatry Clinic due to altered behavior, aggressiveness and presence of delusions and hallucinations. First mental problems in form of a catatonic stupor appeared 6 years ago. Patient has history of earlier abuse of psychoactive substances. A drug test performed at admission was negative. Patient was born in Srebrenica in 1993, he escaped to Tuzla with his mother in July 1995, while father survived escaping on foot. Patient is a first child from his father’s second marriage. The father’s first wife and two minor children were shot by Bosnian Serbs in early 1992. Patient was born a year and a half after death of his siblings and was named after his half-sister. Patient’s father consumed alcohol after the war and was aggressive towards children. In the last two years, patient had frequent hallucinations, he told his parents that voices were telling him to kill his mother and told his father that his children were still alive. Diagnostic processing was performed and diagnosis of schizophrenia was stated. During hospitalization, patient was treated with olanzapine and low doses of haloperidol, along with haloperidol decanoate, which resulted in significant reduction of productive psychotic symptoms. A partial remission is achieved, negative schizophrenic symptoms and cognitive impairments verified by psychological instruments remain.

**Conclusions:**

Case report emphasize transgenerational transmission of trauma: father‘s untreated trauma, alcohol dependency and abuse of the patient in childhood. These findings are important for treatment and therapeutic considerations. Mentalizing is a possible mediator between childhood abuse and negative symptoms. Parental bonding was explored within high expressed emotions theory as a risk factor for relapse to psychosis, especially the “affectionless control” in the parental (mainly father‘s) bonding style. Studies also stated that psychotic patients often show insecure attachment representations. Possible pathway for further analysis could be discussed: a cold parental bonding style leading to experienced emotional neglect and attachment avoidance might be reflected in lower capacity to mentalize. To improve the mentalization capacity, it would be essential to establish a sustainable therapeutic treatment frame.

**Disclosure of Interest:**

None Declared

